# 202. Telehealth-based HCV Treatment Improves Access to Care for Individuals Who Utilize Syringe Service Programs

**DOI:** 10.1093/ofid/ofaf695.074

**Published:** 2026-01-11

**Authors:** Sarah E Rowan, Kevin Kamis, Elizabeth Golding, Theodore Yoder, Sophia Goldin, Meron Haile, Laura Weinberg, Rachel Green, Ruth Kanatser, Jason Haukoos, David L Wyles

**Affiliations:** Denver Public Health, Denver, CO; Denver Health and Hospital Authority, Denver, Colorado; University of Colorado School of Medicine, Aurora, Colorado; University of Colorado School of Medicine, Aurora, Colorado; University of Colorado School of Medicine, Aurora, Colorado; Denver Health and Hospital Authority, Denver, Colorado; Denver Health and Hospital Authority, Denver, Colorado; Denver Health and Hospital Authority, Denver, Colorado; Harm Reduction Action Center, Denver, Colorado; Denver Health and Hospital Authority, Denver, Colorado; Denver Health, Denver, CO

## Abstract

**Background:**

People who inject drugs (PWID) are disproportionately affected by hepatitis C (HCV) but often do not receive treatment. HCV treatment in partnership with syringe service programs (SSPs) is one way to address this disparity. We implemented telehealth-based HCV treatment programs at 3 SSPs in Colorado and evaluated the effect of the programs on annual treatment rates for PWID with HCV in our safety net healthcare system.

**Figure 1.**
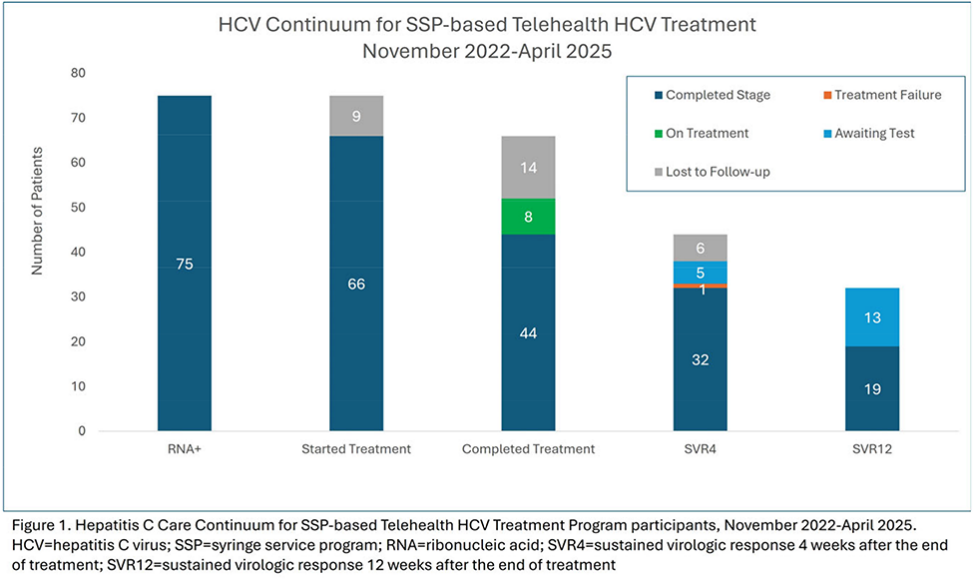
Hepatitis C Care Continuum for SSP-based Telehealth HCV Treatment Program

**Methods:**

In November 2022, we began offering telehealth video consultations for SSP clients in Denver with self-reported HCV, along with on-site phlebotomy and medication storage. A pre-post analysis was completed to compare the proportion of individuals with evidence of HCV and injection drug use that received HCV treatment in our healthcare system in the year prior to the program (clinic-based treatment only) versus during the first year of the program (clinic-based plus SSP-based treatment). After the first year of program implementation, the program was expanded to 2 additional SSPs in Colorado (Boulder and Pueblo). Progression along the HCV care continuum was followed for all SSP program participants enrolled through April 2025.

**Results:**

SSP-based telehealth for HCV treatment offered in addition to standard clinic-based treatment resulted in a higher proportion PWID treated for HCV throughout our healthcare system in the first year of program implementation compared to the year prior to implementation (30.1% [47/156] vs 20.1% [30/149], difference: 10.0%, 95% CI: 3.4% to 19.7%, p=0.04). To date, the SSP-based HCV telehealth program has been operating continuously for 30 months and has enrolled 117 individuals through April 2025. 64% of SSP HCV program participants (75/117) had HCV RNA detected, 88% (66/75) of those with +HCV RNA started treatment, 69% (52/75) completed treatment or were on treatment at the end of the study period, 43% (32/75) had evidence of sustained virologic response 4 weeks after the end of treatment (SVR4) (Figure). One individual experienced treatment failure and was retreated with a second-line regimen.

**Conclusion:**

Telehealth-based HCV treatment at SSPs is a feasible approach to offering HCV treatment and may increase the proportion of PWID who receive HCV treatment in a safety net healthcare system.

**Disclosures:**

All Authors: No reported disclosures

